# Supplementary material for: A History and Atlas of the Human CD4+ T Helper Cell
Source: Biomedicines. 2023 Sep 23;11(10):2608. doi: 10.3390/biomedicines11102608 (PMC10604283; doi:10.3390/biomedicines11102608)
Supplement: Supplementary file 1 [file biomedicines-11-02608-s001.zip › Supplementary Figures S1-S3.pdf]

Supplementary Materials

Jacqueline M. Crater, Daniel C. Dunn, Douglas F. Nixon and Robert L. Furler O'Brien *

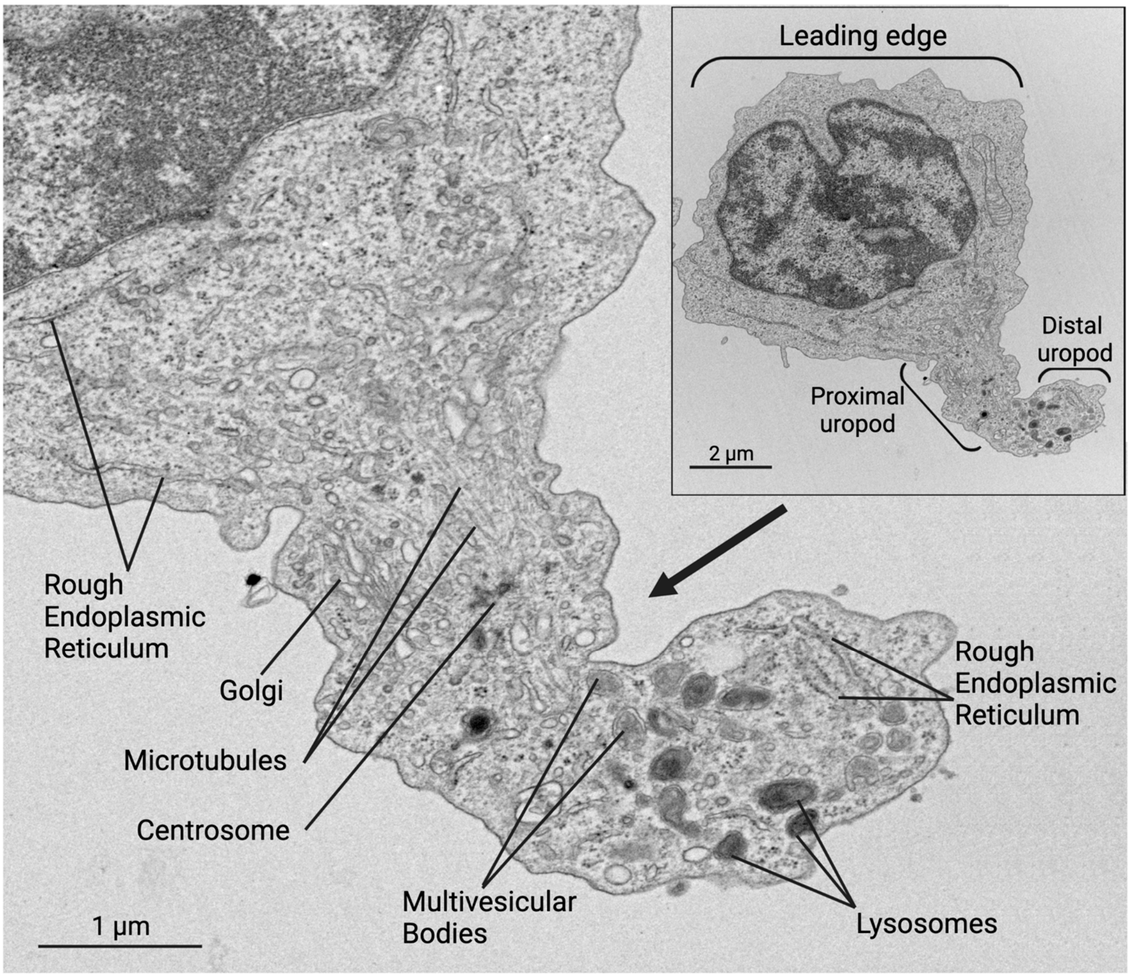

Figure S1. In a migrating CD4+ T cell, the uropod has a high concentration of organelles. These include lysosomes, rough and smooth ER, multivesicular bodies, and Golgi bodies. Most membrane organelles are connected to are localized by microtubules emanating from the centrosome. Created with BioRender.com.

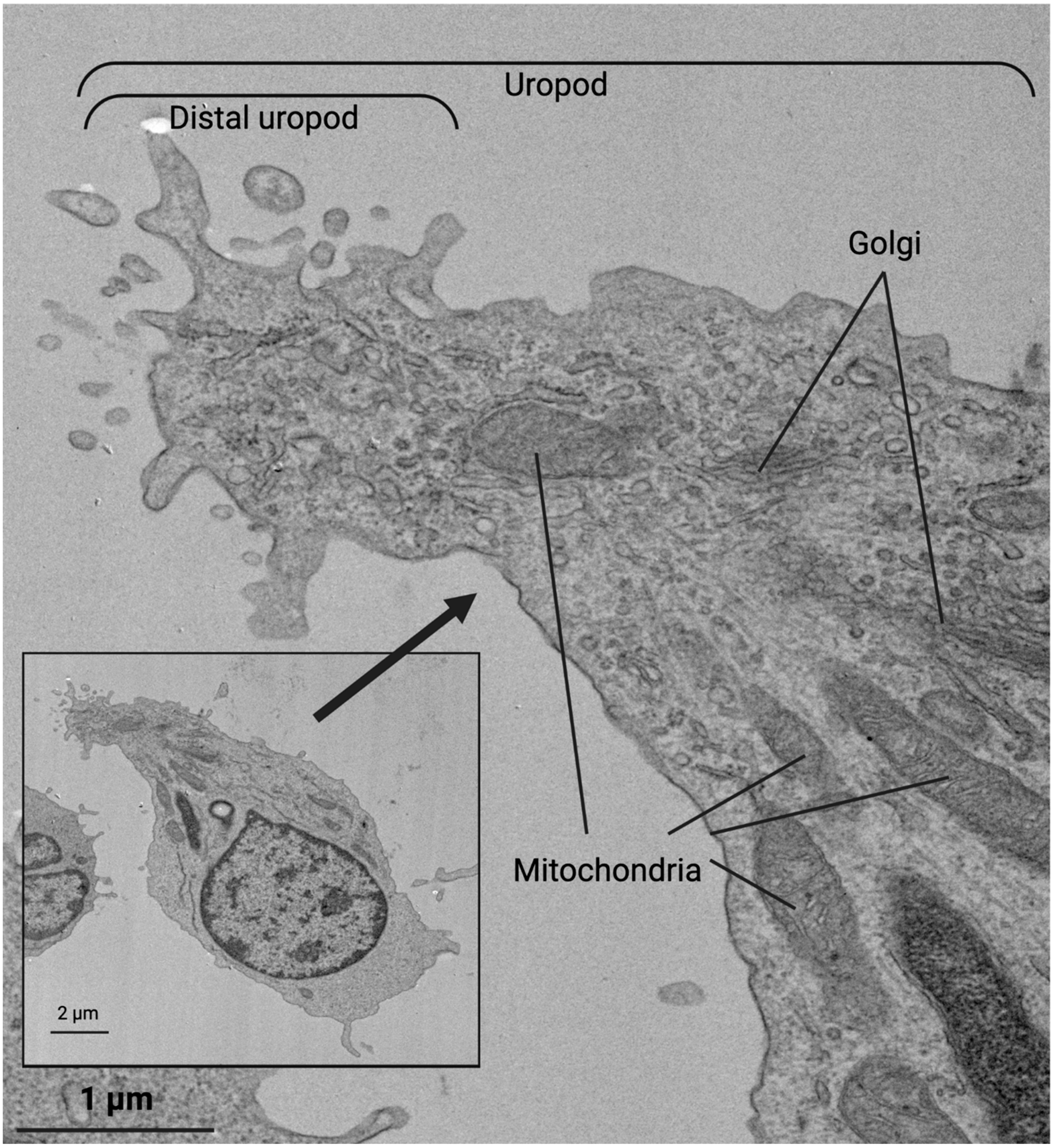

Figure S2. The distal uropod displays different morphology from the rest of the uropod. The distal uropod contains more actin protrusions at the plasma membrane compared to than the proximal uropod. In this image, the mitochondria and Golgi are in the proximal part of the uropod, as organellar degradation and lysosome are often highly concentrated the distal uropod. Created with BioRender.com.

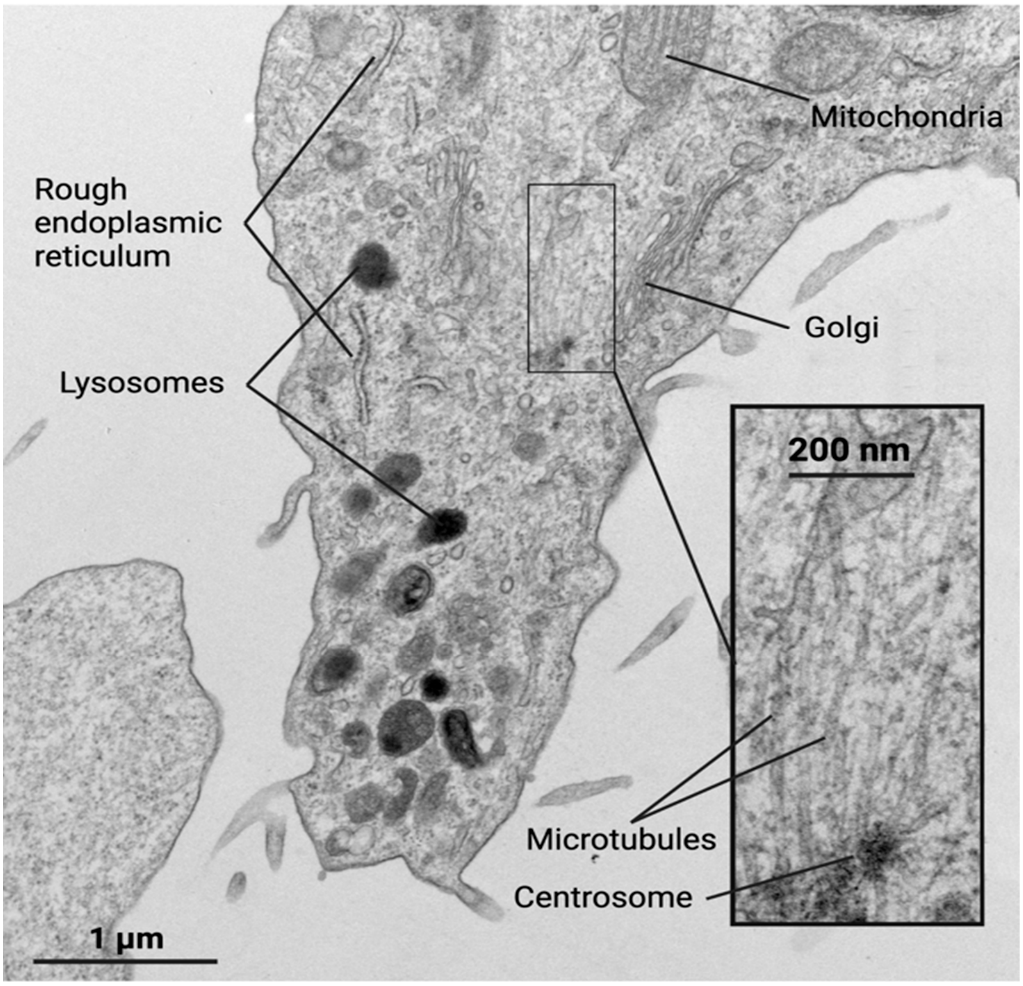

Figure S3. The dense cloud in this TEM section towards the rear of the uropod is the centrosome. Microtubules branching from the centrosome connect to and polarize the organelles within the distal and proximal uropod. Functional mitochondria are located more proximal to the nucleus, while lysosomes are seen in the most distal portion of the uropod, allowing for organellar degradation. Multiple Golgi stacks are located anterior and posterior to the centrosome, which may be useful for guiding polarized secretion. Created with BioRender.com.
